# Considering Cell Therapy Product “Good Manufacturing Practice” Status

**DOI:** 10.3389/fmed.2018.00118

**Published:** 2018-04-30

**Authors:** Patrick Bedford, Juliana Jy, Lucas Collins, Steven Keizer

**Affiliations:** Centre for Commercialization of Regenerative Medicine (CCRM), Toronto, ON, Canada

**Keywords:** cell therapy, clinical trials, regulatory, manufacturing, good manufacturing practices, international perspective

## Introduction

The promise of cell therapies is beginning to be recognized internationally. Cell therapy products (CTPs) are different from other drug products because many aim to be curative, and they are less comprehensively characterizable and more variable, sometimes being manufactured one lot at a time. They have complex mechanisms of action that remain incompletely understood. These unique features have led some regulators to take different approaches to cell therapy regulation in the form of special guidance for navigating regulatory frameworks that were originally intended for pharmaceutical drugs.

A consideration for CTP developers is where to manufacture them, and under what circumstances. With few regulators developing manufacturing requirements specifically for CTPs, highly harmonized regulatory principles of Good Manufacturing Practice (GMP) for pharmaceuticals and biologics apply. However, what people mean when they say they make “GMP grade” CTPs remains ambiguous, and this confusion is exacerbated because most CTPs in early stage development are processed in whole or in part in academic hospital-based settings instead of industrial facilities.

Since GMP guidelines were not originally written with CTPs in mind, the regulatory landscape has been influenced by “multi-level model of practice-driven institutional change” (1). That is to say that, in the absence of prescriptive regulations, the regulated party improvises to meet what they think the regulator expects, and then reorient approaches based on what others can be seen doing. The resulting practices evolve into basic standards of practice that regulators expect the field to follow. This situation compounds confusion about “GMP grade” CTPs because it presents a moving target and creates uncertainty for manufacturing CTPs emerging at the investigational stage of development.

To inject some clarity, stating that GMP is a grade of material is overly simplistic. Instead, GMP should be considered a product-specific assertion based on what is known about a product’s relevant characteristics and a system of ensuring its quality and must be determined on a case-by-case, product-specific basis. This assertion can be substantiated, which may or may not include direct or indirect evidence of third party (e.g., regulator) review.

Others have identified the need for CTP GMP and noted jurisdictional and study phase differences (2–5). From a review of regulatory documents, here we take a basic look at approaches taken in three full ICH member jurisdictions (Canada, the United States, and the European Union) to briefly summarize in (Table 1) similar but different approaches to assessing GMP throughout clinical trial development, and to suggest potential strategies for cell therapy clinical trial product manufacturers.

**Table 1:** Summary table.

	Description	Distinguishing features	“GMP” inspections	International relevance
Canada (Health Canada)	Cell therapy products (CTPs) are considered drugs that must follow Good Manufacturing Practice (GMP) requirements Flexible risk-based approach is taken (i.e., accommodations to GMP may be made, as required, by the technology) More stringent GMP approach throughout clinical trial phases	Establishment license not strictly required for any phase of clinical trials, although strategically necessary by phase 3	Reviewed by Biologics and Genetic Therapies Directorate (pre-market review group) under Division 5—Clinical Trial Application for all phases of clinical trials	No explicit evidence for meeting GMP is provided by Health Canada Implicit evidence of meeting GMP standards is provided in a clinical trial “No Objection Letter”
United States (FDA)	CTPs are considered drugs that must follow GMP and GTP requirements FDA relies on pre-market regulatory professionals to assess phase 1 products for clinical trials during Investigational New Drug (IND) submission reviews, while CT products for phase 2 and 3 require a manufacturing site registration	Establishment license required to manufacture phase 2 and 3 clinical trial products	Phase 1 clinical trial products reviewed under IND submissions by CBER (FDA) Establishment registered with FDA, listed on DECRS	An establishment registration from US FDA indicates GMP compliance for phase 2 or 3 studies (but not phase 1) This strongly supports Health Canada or EMA submissions
European Union (EMA)	Requirements for advanced therapy medicinal products well established, most defined out of the three ICH member jurisdictions under comparison	Manufacturing authorization required for all stages of clinical trial development	Inspections by a competent authority/qualified person (QP) of a member state	A QP site declaration indicates the site complies with EU GMP for all phases of study This strongly supports Health Canada or US FDA submissions

## Canadian Context for Manufacturing CTPs

Canada states that CTPs are drugs that must be manufactured according to GMP requirements aside from specific sample testing and retention requirements, and that “Cell therapies will be held to increasingly stringent manufacturing controls as they are developed from early to late stage clinical trials” (6).

Health Canada’s regulation surrounding cell therapy clinical trial manufacturing requirements is the most flexible and simplest to follow, but it lacks detail. Health Canada’s approach stands out because the regulator does not require manufacturing establishments to register or obtain a license for their manufacturing establishment when they only produce products under *Division 5—Clinical Trial Application* of the *Food and Drug Regulations*. Regulator assessment of cell therapy clinical trial products against GMP principles is performed by Clinical Trial Application reviewers for all stages of clinical trial development. This is done by the Biologics and Genetic Therapies Directorate pre-market review group, who have the authority and specific training to conduct on-site inspections as required, and it does not typically involve Health Canada’s establishment licensing group. As can be seen below, this is different from what is done by the US FDA and European Medicines Agency (EMA), who have compliance experts assess GMP for phase 2 and 3 clinical trial products, and for all clinical trial products, respectively.

Any authorized clinical trial sponsor in Canada claiming to manufacture under GMP is accurately stating what they must do—they must meet all principles of *Division 2—GMPs* that apply to fabricating material for use under *Division 5—Clinical Trial Applications*; however, these establishments will only have evidence of regulatory approval of GMP in the form of a No Objection Letter for a specific clinical trial product manufactured in their facility.

## The United States Context for Manufacturing CTPs

The US FDA relies on pre-market regulatory professionals to assess against GMP as part of a product-specific Investigational New Drug (IND) application for phase 1 studies (i.e., under Section 501(a)(2)(B) of the Food, Drug, and Cosmetic Act). However, these studies are explicitly exempted from GMP regulations described in 21 CFR 211 unless and until they are used (or have previously been used) in phase 2 clinical trials or later, at which time they must Register the site with the US FDA (7). This represents a distinct difference between phase 1 and later phase clinical trials. It can be assumed that in the case of phase 1/2 studies, GMP is measured against more detailed 21 CFR 211 requirements that typically apply for larger phase 2 studies.

The US FDA review of phase 1 studies is like what is done by Health Canada—both are done in the context of an IND application context and rely primarily upon the available data that supports the safety, purity, identification, and strength of the new CTP (3). The US FDA review of manufacturers for later stage study products is more like a European marketing authorization because it requires the US FDA site Registration and inspections to confirm the adherence to GMP standards set forth in 21 CFR 211. A site inspection may still be required by US FDA pre-market reviewer for the assessment of an IND application, if they are unable to determine the risks of the proposed CTP to the patient (3).

Phase 1 IND trial authorization is implicit evidence of meeting US FDA GMP standards, while later phase IND trial evidence of meeting US FDA GMP standards is explicitly found in the form of a site license, which can be searched on a US FDA database.

## The European Context for Manufacturing CTPs

In the European Union (EU), the EMA, has developed *lex specialis* legislation specifically for advanced therapy medicinal products (ATMP). Together with legislation for Medicinal Products for Human Use and Investigational Medicinal Products (IMP), the ATMP regulations stipulate that an IMP “shall be manufactured by applying manufacturing practice which ensures the quality of such medicinal products in order to safeguard the safety of the subject and the reliability and robustness of clinical data generated in the clinical trial (‘good manufacturing practice’)” (8). All IMP trials conducted in a Member State require an application for clinical trial authorization to be submitted to the competent authority of the Member State in which the sponsor plans to conduct the clinical trial (9). The Guidelines for GMP specific to ATMP further specifies that the quality of the imported batch(es) are to be in accordance with the terms of the clinical trial authorization (10). Information on the GMP certification can be accessed on the EudraCT database. Like all drug manufacturers, manufacturers of ATMPs intended to be used as IMPs are required to hold a manufacturing authorization and batches of drug product are required to be certified by a qualified person (QP) (11, 12).

European Medicines Agency manufacturing authorization is issued by a competent authority of a Member State for all investigational products, regardless of the phase of study. This is dependent upon successful completion of an inspection, where the manufacturer demonstrates compliance with GMP principles and guidelines (6, 13) as well as European Union Tissue and Cells Directive requirements (14). The requirement for the manufacture of IMPs in accordance with GMP is further reiterated in the “Guidelines on Good Manufacturing Practice specific to Advanced Therapy Medicinal Products,” newly issued by the European Commission on November 22, 2017 (15). This guideline describes the need for a functional quality system for the manufacturing of investigational ATMPs even during the early phases of clinical development (16).

As evidence of meeting GMP, anyone manufacturing an ATMP for clinical trials in an EU member state will be able to point to their site license in the EudraCT database.

## Opinion

Despite differences in international regulatory approaches to requiring evidence of GMP compliance throughout product development, all manufacturers of CTP clinical trial materials must strive to meet GMP principles. While CTP manufacturing move through academic facilities into industrial facilities they need to meet the same performance-based GMP principles, using the same interpretive flexibility. This is because the safety of clinical trial participants is paramount, and any data developed in clinical trials is inherently more robust when one can be reasonably assured by evidence of the quality of a product that is manufactured consistently. Nevertheless, administratively different ways to assess GMP of CTPs at different phases of clinical study exist internationally and are relevant.

While academic and industry manufacturing facilities are not (and should not be) inspected against different GMP standards or criteria, it makes sense that manufacturing facilities in Canada, the US, and the EU may be inspected differently at each phase of clinical development by different groups of professionals who have a variety of perspectives, expertise, training, and experience. Because of the contextual differences in centralized and country-wide control of GMP regulation, it seems sensible that the stringency for early phase clinical trials is higher for a centralized authority such as the EMA. The risk for different interpretations or standards of the GMP requirements between European member states needs to be managed when different authorities may be looking at clinical trial submissions; this early regulatory requirement can add confidence in product manufacturing. Without the need to address multiple regulatory bodies reviewing manufacturing materials, North American regulators can ease the regulatory burden of obtaining manufacturing site licenses.

Regional differences in regulatory methods in Canada, the US, and the EU shape different approaches, making it possible that each review group will interpret GMP requirements for CTPs from their own unique perspectives. Since one can predict which group of professionals will assess against GMP principles (based on clinical stage of development), one can consider the implications of manufacturing in Canada, the US, or the EU. Assuming results from clinical trials in any ICH member country are equally supportive of later regulatory submissions, we suggest that having premarket cell therapy reviewers assess against GMP for phase 1 or 2 trials is preferable to having facility GMP experts assess cell therapy manufacturing facilities. The former is more likely to consider unique characteristics of CTPs (in general) or a CTP (in particular) than the latter. Without mitigating circumstances, there are practical reasons to choose to manufacture early stage CTPs in Canada (phase 1 or 2) or the US (phase 1), where manufacturing is reviewed against GMP principles by pre-market review groups who are more likely to take a more tailored risk-based approach.

## Author Contributions

PB planned, coordinated, and drafted this document. SK researched and drafted the FDA section. JJ researched and drafted the EMA section. LC revised the document and contributed to the FDA, EMA and Opinion sections.

## Conflict of Interest Statement

The authors declare that the research was conducted in the absence of any commercial or financial relationships that could be construed as a potential conflict of interest.
